# Improving the Journal

**Published:** 1861-01

**Authors:** 


**IMPROVING THE JOURNAL.**—If we had yielded to the thousand and one hints proffered "in fee," for the improvement of our JOURNAL, it would probably have died of inanition years ago; but from the very first we have made it a point to take nobody's advice, to make no attempt to please any one but our supreme self. But we do believe that we have the easiest-pleased set of subscribers in the world; if any thing exceeds it, it is the partiality of our exchanges, exhibited in the fact, that at various times, every single article in a whole number has been copied by some one of them. We do not believe that the same thing can be said of any other monthly publication in the civilized world, where the whole was written by the editor. Sometimes, however, a subscriber suggests to us, in the most chary manner possible, another class of subjects, or the omission of some as not being particularly pertinent to health. The greatest unanimity, however, is in the direction of an inquiry as to the connection between the subject treated and health. On the reverse of the sheet on which we are writing, (those reverse sides of notes, letters, etc., have constituted our entire supply of writing-paper for the JOURNAL since its commencement,) a venerated friend of three-score and ten remarks deprecatingly of our article on "Donation Parties:" "By the way, this subject, although treated by you in an interesting manner, does not fall within the scope of a Journal of Health." We might have inquired, Yankeely, if there was no "connection" between feeding ministers on sham and unusable toys, and moonshine, and starvation! We once gave directions for "doing up" shirt-bosoms neatly. A doctor wanted to know the "connection." We did not think it worth while to impart the desired information at the time. He has since then subsided into invisibility. But there is a very close connection between cleanliness of any portion of our apparel and health. We have often thought of the criticism since, and it has led us to inquire whether it is not every one's duty to make an effort to appear as tidy as possible in the presence of others. The very contemplation of tidiness tends to produce an imitation of it, an ambition for at least an equal amount of it. Who shall not say that the sight of a very poor but tidy child, its clean and well-patched garments, does not on the instant send our thoughts to the home of its mother, with an irrepressible feeling of respect and sympathy for her? We have many a time seen the gentleman, in the "foxy," well-brushed hat, and the thread-bare coat, or neatly darned pantaloons, worn through at the knee.

But our thoughts have gone further, and as this is a gossiping article, they may as well be recorded. Is it not better to hide our deformities on the street and in the drawing-rooms of our friends, and remove from our persons, or cover whatever there may be calculated to excite other than pleasurable feelings? Is it not better to wear a beautiful set of false teeth, such as Allen of Bond street makes, than to appear with unsightly snaggles or with toothless gums; with an artificial limb, than with none at all; with a handsome wig or comely cap, than a bald pate? We ourselves have an involuntary respect for any old man or woman who, through the infirmities of age and the sorrows of time, evidently labors for a tidy personal appearance. It costs them labor, but they prefer it to lazy slovenliness.

Once on a time, long, long ago, when we sometimes admitted articles not original, one of the Sparrowgrass Papers appeared in our pages. A much-respected and worthy lady friend wrote to know the "connection." We thought it was "imminent," direct, a perfect concatenation, for we knew of a case where it caused an unmentionable secession by reason of the vehement cachinnations caused thereby. So with pages 24 and 25.

So, let it suffice once for all to say, that we do not want to be always writing about sickness, disease, and death; about doses, and signs, and symptoms; for actually, the true mode of bringing on a hygienal millennium, for banishing disease from the world as far as may be, is to begin at a point far back, when as yet there is no disease, and do what may be done to inculcate whatever may foster and encourage purity of heart, integrity of character, industry, economy and temperance in practical life; and in every thing promoting cheerfulness, courage, hopefulness and a lofty ambition to excel in the occupation or sphere in which the reader may be placed. Hence the unwavering persistence we have shown in every number since the first, and in almost every page, on an average, in upholding the Sabbath-day, the Bible, our holy religion, its ministers and its friends; and the hour we cease to do the same, may this hand write not another word forever; for in heaven all is purity and goodness, and in heaven alone is there no disease or suffering.

